# Development of the inventory of clinician attitudes about suicide prevention

**DOI:** 10.1111/papt.70092

**Published:** 2026-07-07

**Authors:** Robert J. Cramer, Sarah Spafford, Sam C. Cacace, Skyler D. Prowten, Keyne C. Law, Ryan M. Hill, Ken McDonald, Cindy Charles, Damilola Kolade, Francesca Kassing, Sonyia Richardson, Apryl A. Alexander, Stephanie Renno, Amanda Rowan, Robin Gluck, Chelsea Fiduccia, Justin C. Baker, Shannon Cain, Pedro Gondim, Kairi Kolves, Jacinta Hawgood

**Affiliations:** ^1^ Department of Epidemiology and Community Health & Violence Prevention Center, University of North Carolina at Charlotte Charlotte North Carolina USA; ^2^ University of Oregon Eugene Oregon USA; ^3^ School of Psychology and Clinical Language Sciences, University of Reading Reading UK; ^4^ Seattle Pacific University Seattle Washington USA; ^5^ Department of Psychology, Louisiana State University Baton Rouge Louisiana USA; ^6^ Fraser Health Authority Surrey Canada; ^7^ Rutgers University Newark New Jersey USA; ^8^ Alliant University Fresno California USA; ^9^ School of Social Work, University of North Carolina Chapel Hill North California USA; ^10^ Cohen Veterans Network Stamford Connecticut USA; ^11^ Therapist Development Center Los Angeles California USA; ^12^ Metrocare Services Dallas Texas USA; ^13^ Department of Psychiatry and Behavioral Health, The Ohio State University Wexner Medical Center Columbus Ohio USA; ^14^ Atrium Health Charlotte North Carolina USA; ^15^ Mental Health Academy Fortitude Valley Australia; ^16^ Australian Institute for Suicide Research and Prevention, Griffith University Brisbane Australia

**Keywords:** attitudes, competency, measurement, mental health provider, suicide prevention

## Abstract

**Objectives:**

Mental health providers (MHPs) hold varying attitudes about suicide prevention, and these beliefs can impact personal and client well‐being. To date, suicide prevention attitude measures are limited by appropriate population use, poor psychometrics and a lack of theoretical foundation. The present study rectified a measurement gap in the literature by articulating initial development of the Inventory of Clinician Attitudes about Suicide Prevention (ICASP).

**Design:**

MHPs (*N* = 410) across three countries (United States, Canada and Australia) took part in a cross‐sectional online survey about suicide prevention competencies.

**Methods:**

A community‐engaged convenience sampling approach was used followed by splitting the sample to perform parallel exploratory factor analysis and item response theory analyses.

**Results:**

Analyses yield a four‐factor ICASP with 22 items: (1) Clinician's Approach (*ω* = 0.79); (2) Clinician Avoidance (*ω* = 0.76); (3) Moral Rights (*ω* = 0.84); and (4) Clinician Comfort (*ω* = 0.72). Exploratory findings suggest convergent validity via significant, yet modestly sized, correlations with attitudes glorifying suicide and three elements of compassion fatigue (i.e. compassion satisfaction, burnout and secondary traumatic stress).

**Conclusions:**

The ICASP represents a promising tool for measurement of MHP suicide prevention attitudes in clinical supervision, self‐reflective practice and training evaluation. Findings support portions of the Dynamic Balance Model of MHP suicide prevention attitudes. Future psychometric research directions are discussed.

**Practitioner points**

- Mental health providers hold positive and negative views of suicide prevention practice that can affect care.
- The Inventory of Clinician Attitudes about Suicide Prevention can be used in clinical supervision, self‐reflective practice and training evaluation to enhance suicide care delivery.
- Practitioners may benefit from additional education about suicide prevention attitudes and the Dynamic Balance Model.

Mental health providers (MHPs) routinely encounter clients experiencing suicidal thoughts and behaviours (STBs). For example, an estimated 94.7% of Canadian MHPs have provided care for such clients (Montreuil et al., 2021). Working with clients experiencing STBs often has deleterious consequences on MHPs' emotional health (e.g. anxiety, guilt) and professional practice (e.g. avoidance of clients with STBs, hypervigilance; MacGarry et al., 2022; Sandford et al., 2021). MHP attitudes about suicide prevention may impact clinical decisions and actual or perceived skills (e.g. Groth & Boccio, 2019; Herron et al., 2001; Lee et al., 2016) and may be impacted by clinical training (Jacobson et al., 2012). As a result, efforts to manage one's attitudes or reactions to suicide has been framed as a core skill (Cramer et al., 2013; Rudd et al., 2008) for MHPs. Consequently, an MHP‐specific suicide prevention attitudes measure is valuable for assessing MHP attitudes in supervision and training, as well as investigating how such attitudes impact the use and efficacy of preventive interventions for suicide.

## Mental health provider suicide attitudes within training programmes

There exists a high potential for MHPs' suicide attitudes to impact both clinical care and well‐being. As such, there is an ethical viewpoint that MHPs should manage their suicide‐related attitudes and reactions to bolster the therapeutic alliance and provide sound suicide‐specific services (Barnett & Johnson, 2008; Saigle & Racine, 2018). The wide‐reaching impacts of MHPs' suicide attitudes explain why they are a central feature of existing suicide prevention training models. Burnette et al. (2015) espoused a social‐cognitive model of gatekeeper training for lay persons. Suicide attitudes are highlighted as an explanatory mechanism of how training impacts the intent and actual use of gatekeeper skills. The Core Competency Model of Suicide Prevention Training (CCM; Cramer et al., 2013, 2024) adapted this model to MHPs. Attitudes about suicide, and any relevant suicide‐related subjects, comprise the first essential skills for MHPs within this programme. The Core Competency Model for Corrections (CCM‐C; Cramer et al., 2022; Peiper et al., 2024; Prowten et al., 2025) extended the emphasis on MHPs' suicide attitudes by identifying these beliefs overall, and specific to incarcerated persons engaging in self‐directed violence, as pathways explicating the impact of training on the use of suicide prevention practices.

The centrality of MHP suicide attitudes make them a common training evaluation target. CCM trainings commonly target MHP suicide attitudes through self‐assessment, reflection and interactive training techniques, often resulting in positive shifts post‐training (e.g. Cramer, Long, et al., 2019; La Guardia et al., 2019). Furthermore, Gryglewicz et al. (2020) observed several positive attitude changes (e.g. perceived value of good documentation) among MHPs completing a psychoeducational and role play training. Hawgood et al. (2022) evaluated the Structured Tailored Assessment for Responding to Suicidality Protocol (STARS‐p) training programme; training yielded improved MHP suicide prevention‐related attitudes. These illustrative examples show the ubiquity of MHP suicide attitudes within a training context. However, assessment of attitudes is limited by singular items, internal validity problems or lack of nuanced conceptual development.

## Suicide attitudes: Measures and a conceptual model

Several instruments exist assessing attitudes towards suicide in general populations. Most focus broadly on stereotypical beliefs and stigmatizing attitudes towards suicide rather than attitudes towards engaging in suicide prevention or care. For example, the Suicide Opinion Questionnaire (SOQ; Domino et al., 1980) demonstrated poor psychometric properties including inconsistent factor structure and a wide range in convergent validity and internal consistency across studies (Nicholas et al., 2023). Furthermore, the Stigma of Suicide Scale (SOSS) and its short form (SOSS‐SF) were developed to assess stigmatizing attitudes towards people who die by suicide in the general community (Batterham et al., 2013). While the SOSS may be useful in assessing stigmatizing attitudes in community samples, when used with MHPs, the isolation/depression subscale may function more like a recognition test of suicide warning signs than an index of stigma, calling its internal validity into question in such contexts (Cramer, La Guardia, et al., 2019). Another example is the Attitudes Towards Suicide Scale (ATTS) which showed multidimensional factor structure with low internal consistency within each subscale (Renberg & Jacobsson, 2003). These examples mirror a broader set of general population suicide attitudes measures (see Ghasemi et al., 2015).

A smaller set of instruments attempt to assess attitudes specifically towards suicide prevention, particularly among health care providers. Developed on a combination of MHPs and general health care providers (e.g. primary care), the Attitudes to Suicide Prevention Scale (ASPS) focuses on MHPs' beliefs about the preventability of suicide and their role in prevention (Herron et al., 2001). The ASPS frames suicide prevention narrowly and omits the relational and moral dimensions of suicide care. Developed on nurses, the Suicide Behaviour Attitude Questionnaire (SBAQ) was designed to assess the impact of suicide prevention training (Botega et al., 2005). However, the factor structure has shown poor replication with varying internal consistency across studies (Botega et al., 2007; La Guardia et al., 2019). Developed on a multidisciplinary (e.g. police, health care providers) sample, the Occupational Suicide Attitude Questionnaire (OSAQ) addresses post‐training optimism and organizational support (Ramberg et al., 2021). However, the scale treats suicide prevention attitudes as global constructs and misses vital practice‐focused aspects of care.

Existing suicide‐related attitude measures are limited with respect to population of focus, psychometric properties and theoretical basis. Podlogar et al. (2020) provided fertile theoretical grounding for an MHP‐specific suicide prevention measure. They derived a six‐dimension conceptual Dynamic Balance Model based on MHP interviews and grounded theory analysis. The Dynamic Balance Model assumes that attitudes are driven by the MHP‐client dyad, accounting for the therapeutic setting and health care system regulations. The six dimensions of MHP suicide prevention attitudes were: (1) Understanding suicidality (i.e. the difference between possessing suicide knowledge in general vs. focusing on the specific client), (2) the role of the alliance (i.e. the extent to which an MHP believes the alliance is protective vs. not a guarantee to be helpful), (3) attitudes (i.e. acceptance of the realities of client suicide vs. having a life‐promoting orientation), (4) emotional response (i.e. the extent of worry about or distrust of clients during suicide care), (5) responsibility (i.e. the difference in believing suicide prevention is an MHP's professional, ethical duty vs. respecting the client's autonomy) and (6) focus (i.e. whether the focus of care is specific to suicidal ideation, plan, etc., vs. other factors in a client's clinical picture).

## The present study

The present study fills an important gap by using an established conceptual model to derive an empirical assessment of MHP attitudes towards suicide. This measure is important because it can be used in self‐reflective practice towards MHP self‐care and employed in clinical supervision to monitor trainee progress. Furthermore, in line with competency‐based models of suicide prevention training (e.g. Burnette et al., 2015; Cramer et al., 2024), this instrument has the potential for use in evaluation of training impacts on MHP attitudes about suicide. As such, this study featured initial development of the Inventory of Clinician Attitudes about Suicide Prevention (ICASP). For the sake of ethical research transparency, we acknowledge the exploratory nature of the initial ICASP development. Therefore, we examined the following research questions (RQs) as opposed to a priori hypotheses.

What are the initial ICASP factor structure and reliability?

What are the supplemental psychometric characteristics of the emergent ICASP factor structure? (e.g. item discrimination, subscale validity associations with suicide prevention knowledge, general attitudes about suicide and compassion fatigue)?

## METHODS

### Participants

Table 1 contains demographic information for the full sample. The majority of the sample identified as women (*n* = 342; 83.4%) and possessed licensure (*n* = 336; 82.0%). The pooled sample had an average of 11.74 years of experience (SD = 9.18) as an MHP and 41.87 h of suicide prevention training (SD = 304.78). Demographic information for each subsample (Canadian *n* = 135; American *n* = 230; Australian *n* = 45; Pooled *N* = 410) is summarized in Table S1.

**TABLE 1:** Full sample demographic characteristics.

Demographic	*n* (%)	*M* (SD)
Age (in years)	‐	43.29 (11.57)
*Sex assigned at birth*		
Male	50 (12.2)	‐
Female	313 (76.3)	‐
Prefer not to say	2 (0.5)	‐
Missing	45 (11.0)	
*Gender*		
Agender	1 (0.2)	‐
Genderqueer or genderfluid	1 (0.2)	‐
Man	57 (13.9)	‐
Non‐binary	3 (0.7)	‐
Woman	342 (83.4)	‐
Another gender	3 (0.7)	‐
Prefer not to disclose	3 (0.7)	‐
*Sexual orientation*		
Asexual	10 (2.4)	‐
Aromantic	1 (0.2)	‐
Bisexual	28 (6.8)	‐
Gay	3 (0.7)	‐
Heterosexual/Straight	320 (78.1)	‐
Lesbian	6 (1.5)	‐
Queer	15 (3.7)	‐
Pansexual	9 (2.2)	‐
Another sexual orientation	2 (0.5)	‐
Prefer not to disclose	15 (3.7)	‐
*Discipline*		
Counselling	78 (19.0)	‐
Medicine	6 (1.5)	‐
Nursing	68 (16.6)	‐
Occupational therapy	12 (2.9)	
Peer support/lived experience worker	1 (0.2)	‐
Psychology	77 (18.8)	‐
Social work	136 (33.2)	‐
Student	4 (1.0)	‐
More than one	8 (2.0)	‐
Another discipline	19 (4.6)	‐
*Licensed*		
No	73 (17.8)	‐
Yes	336 (82.0)	‐
Missing	1 (0.2)	
*Student*		
No	364 (88.8)	
Yes	46 (11.2)	
Years of experience	‐	11.74 (9.18)
Hours of suicide prevention training	‐	41.87 (304.78)
Suicide exposure		2.98 (1.45)
*Survey measure scores*		
Literacy of Suicide Scale‐Short Form	‐	10.97 (1.17)
Stigma of Suicide Scale‐Short Form		
Stigma subscale	‐	13.12 (5.00)
Isolation‐depression subscale	‐	15.57 (2.99)
Glorification‐normalization subscale	‐	11.05 (2.94)
Professional quality of life		
Compassion satisfaction subscale	‐	40.57 (5.57)
Burnout subscale	‐	21.73 (5.41)
Secondary traumatic stress subscale	‐	20.65 (5.56)
Socially desirable response set		1.54 (1.39)
Vignette condition		
Scenario 1 (suicidal client)	207 (50.5)	‐
Scenario 2 (non‐suicidal client)	203 (49.5)	‐
Likelihood you would accept vignette client in your private practice?	‐	4.95 (1.42)
Willing to provide services to a suicidal client?	‐	5.13 (1.20)

*Note*: Canadian sample *n* = 135; United States sample *n* = 230; Australian sample *n* = 45; Total sample *N* = 410. Continuous variables shown as mean with standard deviation in parentheses. Categorical variables shown with frequency and percentage in parentheses.

Abbreviations: *M* = Mean; SD = Standard deviation.

### Procedure

MHPs were eligible to participate if they were (1) aged 18 years or older; (2) able to read and speak English; (3) working as an MHP or trainee; and (4) lived in Canada, United States, or Australia. A convenience sampling approach was used. Study recruitment occurred via distribution of Qualtrics e‐survey links to community partners' employees, partners or listserv members. Participants who followed the link completed: (1) the e‐consent form, (2) eligibility screening questions and CAPTCHA verification and (3) the full survey (if eligible). Ineligible participants were redirected to a thank you message. In the primary survey, demographic questions appeared first. Remaining survey measures were randomized to reduce order effects. Participants were presented with a debriefing form upon survey completion. Median survey completion time was 28 min.

Community partners varied by country. Fraser Health Authority (FHA) was our sole Canadian community partner. Canadian participants were required to be affiliated with FHA and were not offered compensation upon survey completion. United States and Australian community partners were a combination of academic training programmes (e.g. United States—UNC Charlotte School of Social Work, Australia—Griffith University) and professional organizations (e.g. United States—APA Division 18 of Psychologists in Public Service, Australia—Mental Health Academy). Additionally, authors partnered with non‐profit health care organizations (e.g. Cohen Veterans Network) for United States survey distribution. All United States participants were given the option to enter a drawing to win 1 of 10 $20 Amazon e‐gift cards. Australian participants were given the option to enter a raffle to win 1 of 10 $20 Amazon e‐gift cards; alternatively, if participants were students at Griffith University, they could opt to receive course credit. All study procedures were approved by multiple university institutional review/ethics boards and community partners' oversight bodies.

### Measures

#### Demographics

All participants were asked to self‐report their age, sex, gender, race, ethnicity, occupational discipline, licensure, student status, years of experience as an MHP, previous training in suicide prevention and history of suicide exposure. Prior suicide exposure was assessed by summing the number of lifetime contacts who attempted suicide: client/patient, acquaintance, friend, family member and other person (possible total of 5). Within each survey, demographic questions and response options were tailored to be culturally appropriate in consultation with in‐country partners. For example, Canadian race and ethnicity response options were consistent with Government of Canada (2025) guidance while those in the United States survey were consistent with United States Census Bureau (2024) guidance. Australian demographics were developed in consultation with community partners.

#### ICASP item development

We followed best practices for steps in item development (DeVellis, 2017). We took three steps: (1) developing the item pool based on a conceptual model; (2) exposing the item pool to expert panel review; and (3) revising scale items based on expert feedback combined with alignment to item development guidelines. Podlogar et al. (2020) developed the dynamic balance model, which articulates six dimensions of MHPs beliefs about working with clients experiencing suicidality. Two dimensions are viewed as opposing ends, with the goal of finding balance or harmony across dimensions in order to provide excellent care. We developed a set of 48 items, with eight falling within each dynamic balance model theme: understanding suicide, emotional responses to the client, the role of the alliance, therapist's responsibility, general attitudes (e.g. life‐orientation) and focus on suicidality. Items were rated on a 5‐point scale, with 0 = *strongly disagree*, 4 = *strongly agree*. The original item pool can be found in Appendix S1.

Our team then solicited interested volunteers for an expert panel composed of suicide prevention expert MHPs and researchers. Individuals were selected based on a combination of literature review by our team, clinical reputation in the field or research expertise within collaborative networks. Of the 12 persons originally contacted with a standardized email invitation, five (co‐authors RMH, KCL, FK, CC and DK) took part as expert panel members. The final panel was diverse with respect to race, sexual orientation and years of suicide prevention research and/or practice experience. Panel members were provided with the full set of 48 ICASP items as well as instructions to provide any feedback on content, wording, cultural relevance and any other matters they deemed appropriate. We intentionally left expert panel feedback open‐ended to maximize the breadth and depth of feedback. Panel members had 1 month to complete their reviews and were compensated with a $10 e‐gift card.

Once reviews were completed, primary study authors (SDP, RJC) integrated feedback into one overall ICASP. We followed guidelines for item development in evaluating the feedback. For example, we retained feedback aligning with item development guidelines like avoiding double‐barrelled phrasing, allowing for some item content overlap, retaining a balanced number of items per underlying dynamic balance theme, simplifying language, limiting emotionally charged phrasing, using panel member recommendations regarding culturally appropriate phrasing and dropping any specialized font (e.g. italics). The final item pool had an approximate 8th grade reading level. Survey items were randomized prior to distribution to participants.

#### Literacy of Suicide Scale‐Short Form (LOSS‐SF)

Suicide‐related knowledge was assessed via the Literacy of Suicide Scale‐Short Form (LOSS‐SF) (Calear et al., 2022). Participants are asked to judge whether the 12‐items are true or false. Correctly answered items are scored as 1 while incorrect items are scored as 0. Correct answers are summed such that higher scores align with greater suicide‐related knowledge. Consistent with Item Response Theory (e.g. Ackerman, 1994) approaches to knowledge test properties, we report LOSS‐SF rates of correct responses, rather than internal consistency, in order to gauge the scale's ability to capture the full range of suicide‐related knowledge. MHP correct response rates to LOSS‐SF questions ranged from 41.7% to 100.0%.

#### Stigma of Suicide Scale‐Short Form (SOSS‐SF)

Stigma towards individuals expressing suicidality was measured via the Stigma of Suicide Scale‐Short Form (SOSS‐SF) (Batterham et al., 2013). Participants are given a list of 16 adjectives that could be used to describe someone who has died by suicide. For each item, participants rate their agreement with the word on a 5‐point scale (1 = *strongly disagree*; 5 = *strongly agree*). The SOSS‐SF is divided into three subscales: stigma (8 items), isolation‐depression (4 items) and glorification‐normalization (4 items). Items within each subscale are summed. Each subscale of the SOSS‐SF demonstrated good to excellent estimated reliability in the pooled sample (Stigma Cronbach's *α* = .90; Isolation‐Depression Cronbach's *α* = .85; Glorification‐Normalization Cronbach's *α* = .84).

#### Professional quality of life (ProQol)

MHPs' levels of compassion fatigue and compassion satisfaction were assessed using the Professional Quality of Life Scale (ProQol) (Stamm, 2010). Participants are asked to rate their agreement with 30 items on a 5‐point Likert scale (1 = *never*; 5 = *very often*). The ProQol contains three subscales: compassion satisfaction (10 items), burnout (10 items) and secondary traumatic stress (10 items). Items are summed within each subscale. The ProQol demonstrated good to excellent estimated reliability in the pooled sample (Compassion Satisfaction *α* = .90; Burnout *α* = .80; Secondary Traumatic Stress *α* = .83).

### Data analysis

#### Data preparation

A total of 699 participants responded to the survey. In accordance with current statistical literature (e.g. Arevalo et al., 2022), multiple steps were taken to ensure data quality. Participants were excluded from analysis if they did not consent to the survey (*n* = 2) or did not meet inclusion criteria based on eligibility screening items (*n* = 80). Next, participants were excluded based on extreme time to survey completion (i.e. < 5th percentile; *n* = 30) or less than 60% of survey completed (*n* = 71). Missing data frequencies ranged from 16.1% to 21.3%. For each survey instrument, there was no evidence of linear response patterns. Participants were then removed if they did not complete any ICASP items (*n* = 106). All data preparation procedures were done using IBM SPSS Statistics (Version 28). The final analysable sample included 410 participants (Canada *n* = 135; USA *n* = 230; Australia *n* = 45).

#### Analytic plan

To evaluate research questions in distinct samples, the data were randomly split evenly into two subgroups (RQ1 *n* = 246; RQ2 *n* = 270) to ensure that the factor structure and item‐level measure refinement were not derived from the same sample.

RQ1: To evaluate RQ1 in subsample 1, a multistep process was conducted. First, to estimate the appropriate number of factors in the ICASP, a parallel exploratory factor analysis (pEFA) was conducted using all 48 original items. Parallel analysis repeatedly resamples the dataset, extracting estimated loadings within and between one and the maximum possible number of factors, providing information regarding fit of each potential factor model with no rotation (Hayton et al., 2004). The resulting fit is also compared to fit of a simulated random normal dataset. Missing data were handled using full information maximum likelihood (Enders, 2001). We examined Eigenvalues greater than one alongside the associated Scree plot, the Very Simple Structure (VSS; Wood et al., 2015) and BassAckward approach (Goldberg, 2006) to identify an optimal number of factors supported by the data. VSS is a method that maximizes the number of items which belong to only one factor, based on a series of complexity criteria (Revelle & Rocklin, 1979). The BassAckward approach uses hierarchical factor structures to compare nested models, examining associations between factors of nested models (Goldberg, 2006), for instance, demonstrating associations between a 6 and 5‐factor model's latent factors and subsequent shifts in substantial item loadings. After the optimal factor number range was identified, we proceeded with item reduction, using the maximum optimal number of factors as a guide. To conduct the pEFA, the R package *psych* was used (Revelle, 2025), and *EFAtools* (Steiner & Grieder, 2020) was used to explore alternative factor structures with the BassAckward approach (Goldberg, 2006).

Item reduction was conducted iteratively, with additional exploratory factor analyses (EFA) conducted after removing items at each step based on unsubstantial standardized loadings (i.e. *λ* < 0.350) on any given factor, and inconsequential communality (i.e. variance in the item explained by all factors in the model less than 0.200) (Yong & Pearce, 2013). With each successive model with the next item removed, the latent factor correlation matrix was reassessed for convergence (*r* > .85), overall model fit, Eigenvalues (*E* > 1.00) and variance explained by the optimal range of factors examined (Brown, 2015; Hayton et al., 2004). Overall model fit was assessed by at least two of three overall fit indices meeting acceptable cut‐offs, including a nonsignificant *χ* ^2^, Comparative Fit Index > 0.90 and Root Mean Square Error of Approximation (RMSEA) < 0.80 (Hu & Bentler, 1999). If the variance explained by a factor dropped to less than 4%, the next smallest number of factor model was used to guide item reduction until all loadings were significant and substantial (see decision‐tree for item reduction steps is provided in Figure S1). Item reduction EFA models were run using the R package *lavaan* (Rosseel, 2012). Once a model that adequately represented a theoretically appropriate number of factors was identified, reliability estimates were calculated using McDonald's *ω* (McDonald, 2013) for subscales. Items with negative loadings in the final EFA model were reverse‐coded.

The final factor structure from subsample 1 was entered as the measurement model in subsample 2, including significant and substantial cross‐loadings (i.e. *λ* > 0.350, *p* < .05). Model fit interpretation was determined as outlined above.

RQ2: In light of poor CFA model fit, to evaluate item‐level characteristics in RQ2, a nominal response model (NRM; Bock, 1972) was used to identify item‐level characteristics for further item reduction. NRMs provide information about item discrimination, pseudo‐difficulty and response likelihood for any given category in the absence of the latent trait within any given respondent (see Bock, 1972), providing detailed information about the latent variable's contribution to participant responding and item variability and permitting informed decision‐making for item reduction (see Edelen & Reeve, 2007) as well as recommendations for future scale refinement. The NRM model was conducted using the *mirt* package (Chalmers, 2012). To assess preliminary validity, subscales emerging from the refined ICASP factor structure were examined for bivariate associations with measures of suicide prevention knowledge, general suicide attitudes and compassion fatigue. Pearson *r* values were used to capture these associations. All data analyses were conducted using RStudio v. 2025.05.0 (RStudio Team, 2024).

## RESULTS

### RQ1: Initial ICASP factor structure and reliability

The result of the pEFA and VSS including all 48 ICASP items suggested between five and eight factors, with Eigenvalues decreasing substantially with more than four factors (see Figure S2), and VSS suggesting a 5‐factor solution (see Figure S3). Five‐ and 6‐factor solutions were compared using the BassAckward method. Results indicated that differences between the 5‐ and 6‐factor solutions were primarily due to addition of cross‐loadings between several factors, with only one item loading on Factor 4 (see Figure S4). As a result, the 5‐factor solution was chosen for further analysis. Factors in this model explained between 12% and 26% of the variance in their respective items, loadings to assigned factors exceeded 0.35, and communalities indicated relevance within the factor model for each retained item (see Table S2).

Reliability coefficients were calculated for each of the five factors, with varying results. The 5‐factor solution was comprised of: (1) *Client Autonomy* (6 items; *λ*_range_ = 0.35–0.85; *ω* = 0.63); (2) *Clinician Discomfort* (7 items [3 reverse‐coded]; *λ*_range_ = −0.39 to 0.76; *ω* = 0.35); (3) *Life‐Promoting Orientation* (5 items; *λ*_range_ = 0.37 to 0.69; *ω* = 0.75); (4) *Therapeutic Alliance* (5 items [1 reverse‐coded]; *λ*_range_ = −0.42 to 0.65; *ω* = 0.61); and (5) *Suicide‐Specific Focus* 3 items (3 items; *λ*_range_ = 0.41 to 0.65; *ω* = 0.56). A confirmatory factor analysis on Sample 2 indicated poor overall model fit (*χ* ^2^(395) = 821.60, *SCR* = 1.061, *p* < .001; rCFI = 0.730; rTLI = 0.683; RMSEA = 0.071; SRMR = 0.092). This, in combination with poor reliability, several ICASP factors signified the need for item‐level analysis to refine the measure.

### RQ2: Supplemental psychometric properties: Scale refinement with item response theory and preliminary subscale validity

Using subsample 2, the 5‐factor NRM (*−LL* = 6676.74; *AIC* = 14,025.48; *BIC* = 15,156.44; *saBIC* = 14,091.74) indicated eight items (items 2, 3, 6, 11, 14, 21, 39 & 45) across the measure were not substantially influenced by any of the latent factors in the model, with communalities < 0.25. In addition, these items presented with poor discrimination and difficulty, and suggested no particular response pattern order, indicating that increasing agreement with these items was not indicative of a greater level of the latent construct for their respective factor. Furthermore, these items largely had minimal variation in responses, with many participants selecting the same response across the sample. Four of the eight problematic items belonged to the *Suicide‐Related Focus* factor, so the factor was removed prior to running the subsequent NRM model.

The 4‐factor NRM with 22 items demonstrated an improvement in overall fit (*−LL* = 4799.11; *AIC* = 10,042.23; *BIC* = 10,789.47; *saBIC* = 10,086.01) compared to the 5‐factor model. Table S3a through S3e depict the ICASP 4‐factor model item factor loadings, response proportions, response category discrimination values, response category scoring coefficients (i.e. pseudo‐difficulty of each response option) and response category attractiveness (i.e. the likelihood of choosing a response when an individual has none of the underlying latent trait). The 22 items demonstrated strong loadings within their respective factors with negligible cross‐loadings on other factors (Table S3a). Where cross‐loading occurred, the item was retained on the factor with stronger theoretical alignment. All but two items demonstrated good communality across factors. Furthermore, most items presented a clear order of response scoring coefficients across response categories, indicating levels of the latent factor were captured ordinally in each response (Bock, 1996), and response category discrimination was reasonable across responses. Only a few items demonstrated unusual response category attractiveness (items 8, 27, 32, 26 and 28), with participants having a high likelihood of choosing responses at the one end of the scale. The proposed 4‐factor model comprises the following factors: (1) *Clinician's Approach* (10 items; *λ*_range_ = 0.36–0.83; *ω* = 0.79); (2) *Clinician Avoidance* (7 items; *λ*_range_ = −0.83 to 0.86; *ω* = 0.76); (3) *Moral Rights* (4 items; *λ*_range_ = 0.69–0.99; ω = 0.84); and (4) *Clinician Comfort* (2 items; *λ*_range_ = 0.95–0.95; *ω* = 0.72). The four ICASP subscales displayed divergence, with small, nonsignificant correlations across factors (see Table S4). Retained items for the 4‐factor ICASP with scoring instructions are in Appendix S2.

As an exploratory analysis, items from the 4‐factor NRM model were summed to create composite scores for each proposed subscale and correlated with existing validity measures capturing suicide prevention knowledge, general suicide attitudes and compassion fatigue (see Table 2). *Clinician Approach* displayed a significant positive association with compassion satisfaction (small effect). *Clinician Avoidance* showed significant positive associations with burnout and secondary traumatic stress, as well as a significant negative association with compassion satisfaction (small effects). *Moral Rights* displayed a significant positive association with glorification‐normalization (small effect). Finally, *Clinician Comfort* displayed a significant positive association with compassion satisfaction as well as significant negative associations with burnout and secondary traumatic stress.

**TABLE 2:** ICASP subscale validity correlations.

ICASP subscale	Clinician approach		Clinician avoidance		Moral rights		Clinician comfort	
	*r*	*p*	*r*	*p*	*r*	*p*	*r*	*p*
LOSS‐SF	.13	.071	.04	.555	−.06	.432	−.01	.860
SOSS‐SF (Stigma)	.02	.794	.04	.601	−.12	.095	−.02	.740
SOSS‐SF (Isolation‐Depression)	.13	.064	.05	.514	−.10	.151	−.01	.859
SOSS‐SF (Glorification‐Normalization)	−.05	.523	−.05	.514	.**28**	**< .0001**	.05	.507
PROQUOL (Compassion Satisfaction)	.**27**	**< .0001**	**−.33**	**< .0001**	−.01	.918	.**32**	**< .0001**
PROQUOL (Burnout)	−.12	.094	.**36**	**< .0001**	.02	.832	**−.18**	.**010**
PROQUOL (Secondary Traumatic Stress)	−.12	.105	.**25**	**< .0001**	−.06	.436	**−.17**	.**018**

*Note*: Bold = statistically significant.

Abbreviations: LOSS‐SF = Literacy of Suicide Scale‐Short Form, SOSS‐SF = Stigma of Suicide Scale‐Short Form.

## DISCUSSION

The present study focused on the initial development of an MHP‐specific suicide prevention attitudes measure. Our approach corrected for several limitations in previous suicide‐related attitude measures, such as poor applicability for use among MHPs (e.g. SOSS; Batterham et al., 2013), poor psychometric properties (e.g. SOQ; Domino et al., 1980) or a lack of nuance resulting from an absence of a sound theoretical background (e.g. SBAQ; Botega et al., 2005). Our multi‐country MHP sample yielded a preliminary assessment tool, the Inventory of Clinician Attitudes about Suicide Prevention (ICASP). The ICASP balances efficiency of 22 items with nuance (i.e. four factors) and acceptable psychometric properties (e.g. retaining subscales with good reliability).

After pEFA and IRT analyses, the ICASP contains four subscales: clinician approach, clinician avoidance, moral rights and clinician comfort. The resulting factor structure aligns well with suicide prevention theoretical literature. Conceptually, all four subscales align with themes contained in the Dynamic Balance Model (Podlogar et al., 2020). *Clinician Approach* comprises two elements. First, the MHP believes it ethical to acknowledge a client's suicidal thoughts and behaviours as valid (as opposed to invalidating the feeling), but communicates positive alternatives, thereby instilling hope. Second, emphasis is placed on the importance of the therapeutic alliance as facilitating good suicide prevention and care. These elements reflect aspects of the dynamic balance model's facets of the role of the alliance and a life‐oriented set of attitudes. *Clinician Avoidance* represents a strong affective, cognitive and behavioural response to client STBs. Higher responses reflect fear, guilt and anxiety about client suicide, alongside devaluing of the therapeutic alliance and an increased desire to refer clients who experience STBs. In this way, this subscale reflects much of the model's emotional response theme subject matter (Podlogar et al., 2020). *Moral Rights* align very distinctly with the client autonomy end of the dynamic balance model's responsibility element, as indicated by its focus on respecting the client's decisions, autonomy and moral rights. Finally, *Clinician Comfort* connotes a sense of mindfulness about and focus on a client's STBs, thereby aligning with a specific focus on suicide in the dynamic balance model.

ICASP subscales also fit well with clinical practice literature. For example, *Clinician Avoidance* acknowledges the clinical reality of negative emotional and professional impacts that providing suicide‐focused care can have on MHPs (e.g. MacGarry et al., 2022; Sandford et al., 2021). Indeed, this subscale even reflects empirical data showing MHPs may avoid engaging with client's experience STBs (e.g. Groth & Boccio, 2019). *Clinician Avoidance* also aligns with clinical ethical calls to monitor one's own experiences with suicide so as not to negatively impact the quality of care (e.g. Barnett & Johnson, 2008; Cramer et al., 2024). Furthermore, along ethical practice lines, *Moral Rights* is consistent with the perspective that MHPs should respect client autonomy and decisions, even in the context of suicide care (Jobes & Barnett, 2025). The moral overlay of this subscale also replicates a moral dimension of suicide attitudes seen in prior scales (e.g. Botega et al., 2005) and physician‐assisted dying literature (e.g. Braverman et al., 2017). Similarly, *Clinician Approach*'s dual focus on the value of the therapeutic alliance and focus on client assets and hope aligns well with contemporary collaborative, person‐centred approaches to suicide risk formulation and safety planning (e.g. Bryan et al., 2024; Hawgood et al., 2022). Correspondingly, *Clinician Comfort* taps a unique aspect of MHP attitudes that comports well with mindful, central focus on suicide (as opposed to underlying or associated factors) in treatment (e.g. Gysin‐Maillart et al., 2016; Jobes, 2023).

Secondarily, we explored correlates of MHP suicide prevention attitudes, namely, suicide prevention knowledge, general suicide attitudes and compassion fatigue (i.e. satisfaction, burnout and secondary trauma; Stamm, 2010). The most meaningful ICASP associations were with the glorification‐normalization aspect of general suicide attitudes and all three facets of compassion fatigue (compassion satisfaction, burnout and secondary traumatic stress) (see Table 2). *Moral Rights* was positively associated with a tendency to glorify or normalize the experience of STBs. The glorification of suicide suggests that people experiencing STBs are seen as strong, noble, dedicated or brave (Batterham et al., 2013). It is therefore intuitive to see that an MHP believing that such patients may possess these qualities may have a tendency to respect a client's wishes and decisions, even to end their own life. Further work is needed to understand this complex association, however. For example, a natural question arises whether context may impact the strength of the relationship? That is, does MHP glorification of suicide and *Moral Rights* differ in contexts such as inpatient versus outpatient settings, or otherwise healthy versus terminal diagnosis/physician assisted dying request clinical scenarios?

Regarding MHP compassion fatigue, *Clinician Approach*, *Avoidance* and *Comfort* displayed meaningful associations. *Clinician Approach* and *Clinician Comfort* were positively, and *Clinician Avoidance* negatively, linked with *Compassion Satisfaction*. Furthermore, *Clinician Avoidance* was positively associated with, and *Clinician Comfort* negatively linked to, burnout and secondary stress. Wholistically, MHP attitudes and compassion fatigue appear interconnected, a pattern consistent with broader literature acknowledging the negative cognitive, emotional and professional impacts of suicide prevention work (e.g. MacGarry et al., 2022; Sandford et al., 2021). It further raises the importance of MHP suicide‐related attitudes as an important aspect of suicide prevention training and ethical practice. The CCM training approach, for instance, highlights clinician self‐management skills such as managing one's suicide‐related attitudes and engaging in self‐care (Cramer et al., 2013, 2024). Doing so in training and supervision contexts may help delay or prevent clinician compassion fatigue. Moreover, self‐care plans, including healthy activities and a consultation network, have been argued to be an important ethical practice for MHPs (Barnett & Homany, 2022). Future work may focus on developing a unique MHP programme that integrates attitudinal assessment, competency‐based training and self‐care planning. Likewise, additional longitudinal MHP research is needed to understand the directionality and causal mechanisms linking attitudes and compassion fatigue.

The ICASP represents a promising tool to capture MHP suicide attitudes. Such tools can be used in self‐reflective practice, clinical consultation and supervision and training evaluation. However, further ICASP assessment work is necessary (see Appendix S2 for the ICASP for future use). For example, future MHP samples can be used to (dis)confirm the factor structure and test characteristics that may influence the instrument's function. Such MHP characteristics include, but are not limited to, extent of prior training and clinical experience, discipline, personal suicide exposure and experience with physician assisted dying. ICASP psychometric work should also examine consistency over time and sensitivity to changes in response to established trainings (e.g. Gryglewicz et al., 2020; Peiper et al., 2024).

Strengths of the present investigation include use of an expert panel, multi‐country design, theoretical model and rigorous approach to data quality. The present study also features several important limitations cautioning conclusions and reinforcing the need for further empirical work. Generalizability was restricted, especially with respect to MHP gender (e.g. only 14% men), career stage (e.g. only 11% students) and years of experience. We also did not fully assess key MHP characteristics (e.g. race/ethnicity) due to ethical and cultural considerations. Validity analyses were also limited to cross‐sectional assessment of MHP characteristics. Moving forward, the ICASP should be tested for criterion validity, such as client perceptions of satisfaction, therapeutic alliance and clinical outcomes. It should also be investigated in relation to other measures of suicide attitudes to establish construct validity (e.g. discriminant validity) and incremental validity in clinically oriented outcomes.

## AUTHOR CONTRIBUTIONS

**Robert J. Cramer:** Conceptualization; investigation; writing – original draft; writing – review and editing; methodology; project administration; supervision; resources. **Sarah Spafford:** Conceptualization; investigation; writing – original draft; writing – review and editing; methodology. **Sam C. Cacace:** Supervision; data curation; formal analysis; validation; visualization; writing – original draft. **Skyler D. Prowten:** Writing – original draft; writing – review and editing; investigation; methodology; visualization; formal analysis. **Keyne C. Law:** Investigation; writing – original draft; methodology; writing – review and editing. **Ryan M. Hill:** Investigation; writing – original draft; writing – review and editing. **Ken McDonald:** Writing – original draft; investigation; methodology; conceptualization; project administration. **Cindy Charles:** Investigation; methodology; writing – review and editing. **Damilola Kolade:** Writing – original draft; writing – review and editing; investigation; methodology. **Francesca Kassing:** Writing – original draft; writing – review and editing; investigation; methodology. **Sonyia Richardson:** Investigation; methodology. **Apryl A. Alexander:** Investigation; methodology; writing – review and editing. **Stephanie Renno:** Investigation; methodology; writing – review and editing. **Amanda Rowan:** Investigation; methodology; writing – review and editing. **Robin Gluck:** Investigation; methodology; writing – review and editing. **Chelsea Fiduccia:** Investigation; methodology; writing – review and editing. **Justin C. Baker:** Investigation; methodology; writing – review and editing. **Shannon Cain:** Investigation; methodology; writing – review and editing. **Pedro Gondim:** Investigation; methodology; writing – review and editing. **Kairi Kolves:** Investigation; writing – review and editing; methodology; data curation. **Jacinta Hawgood:** Project administration; data curation; investigation; methodology.

## FUNDING INFORMATION

Authors received no funding for this study.

## CONFLICT OF INTEREST STATEMENT

The authors declare no conflicts of interest.

## ETHICS STATEMENT

This study was conducted in accordance with APA ethics and was approved by three ethics boards.

## Supporting information


**Table S1:** Sample demographic characteristics of participants by country.
**Table S2:** pEFA factor loadings for 5‐factor ICASP solution.
**Table S3a–d:** ICASP 4‐factor NRM model item factor loadings, response proportions, response category discrimination values, response category scoring coefficients, and response category attractiveness.
**Table S3a:** ICASP item factor loadings.
**Table S3b:** ICASP item response proportions.
**Table S3c:** ICASP item category discrimination values.
**Table S3d:** ICASP item response category scoring coefficients.
**Table S3e:** ICASP item response category attractiveness.
**Table S4:** Factor correlations for ICASP four‐factor solution.
**Figure S1:** EFA model item reduction decision tree.
**Figure S2:** Parallel EFA Scree plot comparing observed, simulated and resampled data.
**Figure S3:** Methods for deriving number of factors.
**Figure S4:** Comparison of 5‐ and 6‐factor solutions: Initial with all items and second run with items dropped.
**Appendix S1:** Initial ICASP item pool.
**Appendix S2:** Final ICASP with scoring instructions.

## Data Availability

The data that support the findings of this study are available from the corresponding author upon reasonable request.
