# Supplementary material for: Development of the inventory of clinician attitudes about suicide prevention
Source: Psychol Psychother. 2026 Jul 7;99(4):1519–35. doi: 10.1111/papt.70092 (PMC13749270; doi:10.1111/papt.70092)
Supplement: Supplementary file 1 [file PAPT-99-1519-s001.docx]

**Online Supplement**

**Table 1.** *Sample Demographic Characteristics of Participants by Country.*

| Demographic | | Canada | | United States | | Australia | |
| --- | --- | --- | --- | --- | --- | --- | --- |
|  |  | *n* (*%*) | *M* (*SD*) | *n* (*%*) | *M* (*SD*) | *n* (*%*) | *M* (*SD*) |
| Age (in years) | | - | 43.67 (10.31) | - | 43.62 (11.73) | - | 40.44 (14.00) |
|  | Sex assigned at birth |  |  |  |  |  |  |
|  | Male | 19 (14.1) | - | 31 (13.5) | - | NA | - |
|  | Female | 115 (85.2) | - | 198 (86.1) | - | NA | - |
|  | Prefer not to say | 1 (0.7) | - | 1 (0.4) | - | NA | - |
| Gender | |  |  |  |  |  |  |
| Agender | | 1 (0.7) | - | - | - | NA | - |
| Genderqueer or genderfluid | | - | - | 1 (0.4) | - | NA | - |
| Man | | 19 (14.1) | - | 32 (13.9) | - | 6 (13.3) | - |
| Nonbinary | | - | - | 3 (1.3) | - | - | - |
| Woman | | 114 (84.4) | - | 190 (82.6) | - | 38 (84.4) | - |
| Another gender | | - | - | 2 (0.9) | - | 1 (2.2) | - |
| Prefer not to disclose | | 1 (0.7) | - | 2 (0.9) | - | - | - |
| Canadian Ethnicity | |  |  |  |  |  |  |
| Indigenous and/or First Nations | | 3 (2.2) | - | NA | - | NA | - |
| Métis | | 2 (1.5) | - | NA | - | NA | - |
| Prefer not to answer | | 1 (0.7) | - | NA | - | NA | - |
| Not Indigenous, First Nations, or Métis | | 129 (95.6) | - | NA | - | NA | - |
| Race/Ethnicity | |  |  |  |  |  |  |
| Black or of African descent (African, Afro-Caribbean, African Canadian) | | 2 (1.5) | - | NA | - | NA | - |
| East Asian (Chinese, Korean, Japanese, Taiwanese) | | 1 (0.7) | - | NA | - | NA | - |
| Latino (Latin American, Hispanic descent) | | 1 (0.7) | - | NA | - |  | - |
| Middle Eastern (Arab, Persian, Afghan, Egyptian, Iranian, Lebanese, Turkish, Kurdish, West Asian) | | 23 (17.0) | - | NA | - | NA | - |
| South Asian (Indian, Pakistani, Bangladeshi, Sri Lankan, Indo-Caribbean) | | 3 (2.2) | - | NA | - | NA | - |
| Southeast Asian (Filipino, Vietnamese, Cambodian, Thai, Indonesian) | | 4 (3.0) | - | NA | - | NA | - |
| White (Western and Eastern European) | | 89 (65.9) | - | NA | - | NA | - |
| American Indian, Alaska Native, or Indigenous | | NA | - | 4 (1.7) | - | NA | - |
| Asian or Asian American | | NA | - | 17 (7.4) | - | NA | - |
| Black or African American | | NA | - | 23 (10.0) | - | NA | - |
| Latino/e or Spanish Origin | | NA | - | 16 (7.0) | - | NA | - |
| Native Hawaiian or Pacific Islander | | NA | - | 1 (0.4) | - | NA | - |
| White/European American | | NA | - | 152 (66.1) | - | NA | - |
| Biracial or Multiracial | | 5 (3.7) | - | 9 (3.9) | - | NA | - |
| Unsure | | 1 (0.7) | - | - | - | - | - |
| Asian | | NA | - | NA | - | 1 (2.2) | - |
| Australian | | NA | - | NA | - | 28 (62.2) | - |
| English | | NA | - | NA | - | 2 (4.4) | - |
| German | | NA | - | NA | - | 2 (4.4) | - |
| Greek | | NA | - | NA | - | 1 (2.2) | - |
| Indian | | NA | - | NA | - | 1 (2.2) | - |
| Italian | | NA | - | NA | - | 1 (2.2) | - |
| Scottish | | NA | - | NA | - | 1 (2.2) | - |
| Another race or ethnicity | | - | - | 5 (2.2) | - | 8 (17.8) | - |
| Prefer not to disclose | | 6 (4.4) | - | 3 (1.3) | - | - | - |
| Sexual orientation | |  |  |  |  |  |  |
| Asexual | | 6 (4.4) | - | 4 (1.7) | - | NA | - |
| Aromantic | | - | - | 1 (0.4) | - | NA | - |
| Bisexual | | 4 (3.0) | - | 19 (8.3) | - | 5 (11.1) | - |
| Gay | | - | - | 3 (1.3) | - | - | - |
| Heterosexual/Straight | | 115 (85.2) | - | 173 (75.2) | - | 32 (71.1) | - |
| Lesbian | | 2 (1.5) | - | 4 (1.7) | - | - | - |
| Queer | | - | - | 12 (5.2) | - | 3 (6.7) | - |
| Pansexual | | 2 (1.5) | - | 7 (3.0) | - | - | - |
| Another sexual orientation | | - | - | 3 (1.3) | - | NA | - |
| Prefer not to disclose | | 6 (4.4) | - | 4 (1.7) | - | 5 (11.1) | - |
| Education | |  |  |  |  |  |  |
| Grade 12 or less | | NA | - | NA | - | 8 (8.9) | - |
| Diploma | | 19 (14.1) | - | 1 (0.4) | - | NA | - |
| TAFE | | NA | - | NA | - | 2 (4.4) | - |
| Undergraduate degree | | 51 (37.8) | - | 3 (1.3) | - | NA | - |
| Master’s degree | | 58 (43.0) | - | 177 (77.0) | - | NA | - |
| University/postgraduate | | NA | - | NA | - | 37 (82.2) | - |
| Doctoral degree | | 7 (5.2) | - | 49 (21.3) | - | 2 (4.4) | - |
| Discipline | |  |  |  |  |  |  |
| Counseling | | 26 (19.3) | - | 52 (22.6) | - | NA | - |
| Medicine | | 6 (4.4) | - | - | - | - | - |
| Nursing | | 58 (43.0) | - | - | - | 10 (22.2) | - |
| Occupational Therapy | | 10 (7.1) | - | - | - | 2 (4.4) | - |
| Peer Support/Lived Experience Worker | | NA | - | NA | - | 1 (2.2) | - |
| Psychiatry | | NA | - | 1 (0.4) | - | NA | - |
| Psychology | | 1 (0.7) | - | 58 (25.2) | - | 18 (40.0) | - |
| Social Work | | 25 (18.5) | - | 104 (45.2) | - | 7 (15.6) | - |
| More than one | | - | - | 7 (3.0) | - | 1 (2.2) | - |
| Another discipline | | 9 (6.7) | - | 8 (3.5) | - | 6 (13.3) | - |
| Licensed | |  |  |  |  |  |  |
| No | | 23 (17.0) | - | 36 (15.7) | - | 14 (31.1) | - |
| Yes | | 111 (82.2) | - | 194 (84.4) | - | 31 (68.9) | - |
| Missing | | 1 (0.7) | - | - | - | - | - |
| Student | |  |  |  |  |  |  |
| No | | 132 (97.8) | - | 212 (92.2) | - | 41 (91.1) |  |
| Yes | | 3 (2.2) | - | 18 (7.8) | - | 4 (8.9) |  |
| Years of Experience | | - | 13.47 (9.44) | - | 11.52 (8.55) | - | 7.73 (10.25) |
| Hours of Suicide Prevention Training | | - | 7.73 (10.25) | - | 60.36 (403.11) | - | 21.76 (35.6) |
| Suicide Exposure | | - | 2.81 (1.42) | - | 3.01 (1.48) | - | 3.07 (1.36) |
| Survey Measures | |  |  |  |  |  |  |
| Literacy of Suicide Scale-Short Form | | - | 10.95 (1.28) | - | 10.98 (1.11) | - | 11.00 (1.13) |
| Stigma of Suicide Scale-Short Form | |  |  |  |  |  |  |
| Stigma Subscale | | - | 13.41 (5.17) | - | 12.82 (4.76) | - | 13.77 (5.62) |
| Isolation-Depression Subscale | | - | 15.75 (2.61) | - | 15.56 (3.13) | - | 15.11 (3.31) |
| Glorification-Normalization Subscale | | - | 11.00 (2.85) | - | 11.10 (3.04) | - | 10.98 (2.69) |
| Professional Quality of Life | |  |  |  |  |  |  |
| Compassion Satisfaction Subscale | | - | 39.29 (5.09) | - | 41.51 (5.78) | - | 39.66 (5.02) |
| Burnout Subscale | | - | 23.17 (4.99) | - | 21.10 (5.71) | - | 20.59 (4.21) |
| Secondary Traumatic Stress Subscale | | - | 22.06 (6.02) | - | 20.04 (5.23) | - | 19.46 (4.97) |
| Socially Desirable Response Set | | - | 1.77 (1.37) | - | 1.40 (1.40) | - | 1.54 (1.35) |
| Vignette Condition | |  |  |  |  |  |  |
| Scenario 1 (suicidal client) | | 67 (49.6) | - | 119 (51.7) | - | 21 (46.7) | - |
| Scenario 2 (non-suicidal client) | | 68 (50.4) | - | 111 (48.3) | - | 24 (53.3) | - |
| Likelihood you would accept vignette client in your private practice? | | - | 4.88 (1.34) | - | 4.95 (1.50) | - | 5.18 (1.23) |
| Willing to provide services to a suicidal client? | | - | 5.10 (1.16) | - | 5.05 (1.27) | - | 5.58 (0.84) |

*Note. M*=Mean; *SD=*Standard deviation; Canadian sample *n*=135; U.S. sample *n*=230; Australia sample *n*=45; Total sample *N*=410. NA=Not applicable as response option was not included in country-specific demographic section. Continuous variables shown as mean with standard deviation in parentheses. Categorical variables shown with frequency and percentage in parentheses.

**Table 2.** *pEFA Factor Loadings for 5-Factor ICASP Solution.*

| ICASP Item | F1 λ | F2 λ | F3 λ | F4 λ | F5 λ | Communality |
| --- | --- | --- | --- | --- | --- | --- |
| 8 | 0.13 | **-0.67** | 0.12 | 0.05 | 0.00 | **0.49** |
| 13 | -0.05 | **0.62** | -0.11 | -0.02 | 0.16 | **0.43** |
| 17 | 0.00 | **0.51** | -0.06 | -0.02 | 0.17 | **0.29** |
| 22 | 0.14 | 0.31 | 0.31 | 0.16 | 0.16 | **0.29** |
| 27 | 0.12 | **-0.48** | 0.25 | 0.07 | -0.08 | **0.31** |
| 29 | -0.04 | **0.61** | 0.22 | 0.00 | -0.13 | **0.46** |
| 33 | 0.19 | **0.76** | 0.17 | 0.04 | -0.04 | **0.63** |
| 38 | 0.21 | **-0.39** | 0.30 | -0.12 | 0.05 | **0.25** |
| 3 | 0.28 | -0.12 | -0.19 | 0.03 | 0.26 | **0.21** |
| 6 | 0.29 | 0.13 | -0.02 | -0.27 | 0.32 | **0.25** |
| 15 | **0.72** | 0.02 | -0.14 | 0.00 | 0.03 | **0.57** |
| 16 | **0.85** | -0.01 | -0.09 | 0.01 | -0.03 | **0.75** |
| 19 | **0.72** | 0.01 | 0.00 | -0.02 | -0.03 | **0.52** |
| 32 | **0.58** | 0.00 | 0.14 | -0.04 | -0.02 | **0.33** |
| 45 | **0.35** | -0.09 | 0.25 | 0.13 | 0.04 | **0.22** |
| 18 | -0.05 | -0.03 | **0.42** | 0.13 | 0.20 | **0.28** |
| 20 | -0.13 | -0.05 | **0.50** | 0.14 | -0.02 | **0.34** |
| 26 | -0.19 | -0.03 | **0.69** | -0.05 | 0.02 | **0.54** |
| 28 | -0.07 | 0.19 | **0.61** | 0.07 | -0.07 | **0.48** |
| 42 | 0.11 | -0.01 | **0.37** | 0.16 | 0.11 | **0.22** |
| 4 | **0.36** | -0.06 | -0.01 | 0.32 | -0.30 | **0.37** |
| 11 | -0.10 | 0.01 | -0.09 | **0.65** | 0.00 | **0.39** |
| 23 | 0.02 | 0.07 | 0.04 | **0.65** | 0.12 | **0.46** |
| 24 | 0.11 | -0.07 | 0.20 | **0.50** | 0.02 | **0.38** |
| 34 | 0.06 | -0.07 | -0.06 | **0.39** | 0.05 | 0.16 |
| 37 | 0.01 | 0.06 | 0.00 | **-0.42** | 0.18 | **0.21** |
| 2 | -0.02 | -0.02 | -0.03 | 0.19 | **0.44** | **0.23** |
| 14 | -0.11 | 0.08 | 0.13 | 0.29 | 0.21 | **0.20** |
| 21 | -0.04 | -0.02 | 0.07 | 0.10 | **0.65** | **0.46** |
| 39 | -0.12 | -0.02 | -0.07 | -0.20 | **0.41** | **0.24** |

*Note.* **Bold** font = Substantial factor loading (item retained); pEFA = Parallel exploratory factor analysis

*λ* = Factor loading; F = Factor; ICASP = Inventory of Clinician Attitudes about Suicide Prevention.

**Tables 3a to 3d.** *ICASP 4-Factor NRM Model Item Factor loadings, Response Proportions,*

*Response Category Discrimination Values, Response Category Scoring Coefficients, and Response Category Attractiveness*

*Table 3a ICASP Item Factor loadings*

| ICASP Item | F1 λ | F2 λ | F3 λ | F4 λ | Communality |
| --- | --- | --- | --- | --- | --- |
| 8 | 0.06 | 0.08 | 0.02 | **0.95** | **1.00** |
| 13 | 0.09 | **0.74** | -0.02 | 0.12 | **0.61** |
| 17 | -0.09 | **0.69** | -0.09 | 0.29 | **0.69** |
| 22 | -0.34 | **0.49** | 0.24 | 0.19 | **0.48** |
| 27 | 0.06 | 0.09 | 0.02 | **0.95** | **1.00** |
| 29 | -0.10 | **0.86** | -0.07 | 0.04 | **0.79** |
| 33 | -0.32 | **0.86** | -0.06 | 0.21 | **0.99** |
| 38 | -0.31 | **-0.83** | -0.03 | -0.12 | **0.85** |
| 15 | -0.13 | 0.00 | **0.94** | -0.03 | **0.94** |
| 16 | -0.05 | 0.09 | **0.99** | -0.05 | **0.99** |
| 19 | -0.03 | -0.03 | **0.93** | 0.04 | **0.87** |
| 32 | 0.19 | **-0.57** | **0.69** | 0.21 | **0.85** |
| 18 | **0.79** | -0.18 | -0.02 | 0.29 | **0.91** |
| 20 | **0.54** | -0.02 | -0.18 | 0.20 | **0.46** |
| 26 | **0.75** | 0.09 | -0.30 | 0.26 | **0.91** |
| 28 | **0.83** | -0.33 | -0.11 | 0.03 | **0.88** |
| 42 | **0.80** | 0.01 | -0.08 | 0.08 | **0.71** |
| 4 | **0.53** | **0.54** | 0.27 | -0.17 | **0.48** |
| 23 | **0.36** | 0.16 | 0.09 | -0.06 | 0.14 |
| 24 | **0.44** | 0.24 | 0.03 | 0.01 | **0.24** |
| 34 | **0.39** | 0.10 | 0.08 | -0.03 | 0.15 |
| 37 | **0.65** | **0.49** | 0.20 | -0.01 | **0.62** |

*Note.* **Bold** font = Substantial factor loading (item retained); *λ* = Factor loading; F = Factor;

ICASP = Inventory of Clinician Attitudes about Suicide Prevention.

*Table 3b. ICASP Item Response Proportions*

|  |  |  | Proportions |  |  |
| --- | --- | --- | --- | --- | --- |
| ICASP Item | 0 | 1 | 2 | 3 | 4 |
| 8 | 0.23 | 0.48 | 0.03 | 0.03 | 0.01 |
| 13 | 0.20 | 0.30 | 0.20 | 0.06 | 0.02 |
| 17 | 0.27 | 0.31 | 0.13 | 0.06 | 0.01 |
| 22 | 0.06 | 0.24 | 0.23 | 0.22 | 0.04 |
| 27 | 0.23 | 0.48 | 0.07 | NA | NA |
| 29 | 0.06 | 0.17 | 0.20 | 0.27 | 0.10 |
| 33 | 0.09 | 0.23 | 0.21 | 0.23 | 0.03 |
| 38 | 0.32 | 0.40 | 0.07 | NA | NA |
| 15 | 0.06 | 0.17 | 0.33 | 0.19 | 0.04 |
| 16 | 0.06 | 0.19 | 0.34 | 0.18 | 0.02 |
| 19 | 0.03 | 0.12 | 0.38 | 0.20 | 0.05 |
| 32 | 0.01 | 0.07 | 0.16 | 0.38 | 0.17 |
| 18 | 0.00 | 0.05 | 0.27 | 0.32 | 0.14 |
| 20 | 0.02 | 0.04 | 0.16 | 0.37 | 0.20 |
| 26 | 0.00 | 0.02 | 0.16 | 0.36 | 0.25 |
| 28 | 0.00 | 0.03 | 0.14 | 0.47 | 0.14 |
| 42 | 0.00 | 0.03 | 0.09 | 0.46 | 0.19 |
| 4 | 0.02 | 0.04 | 0.10 | 0.33 | 0.29 |
| 23 | NA | 0.03 | 0.14 | 0.43 | 0.19 |
| 24 | 0.00 | 0.00 | 0.04 | 0.38 | 0.36 |
| 34 | 0.01 | 0.06 | 0.17 | 0.45 | 0.11 |
| 37 | 0.11 | 0.53 | 0.15 | NA | NA |

*Note.* F = Factor; ICASP = Inventory of Clinician Attitudes about Suicide Prevention.

*Table 3c. ICASP Item Category Discrimination Values*

| ICASP Item | 0 | 1 | 2 | 3 |
| --- | --- | --- | --- | --- |
| 8 | -10.863 | 3.673 | -8.547 | 15.448 |
| 13 | -0.498 | 0.544 | -0.203 | -0.046 |
| 17 | -0.447 | 0.698 | -0.335 | 0.072 |
| 22 | -0.118 | 0.595 | 0.109 | 0.096 |
| 27 | -22.398 | 7.715 | -17.439 | 31.579 |
| 29 | -0.544 | 1.062 | -0.294 | -0.215 |
| 33 | -1.562 | 4.852 | -1.218 | -0.184 |
| 38 | -1.756 | 1.175 | -0.515 | -0.075 |
| 15 | 0.130 | -0.447 | -2.364 | -0.641 |
| 16 | 1.026 | -1.246 | -5.960 | -1.519 |
| 19 | 0.248 | -0.155 | -1.536 | -0.555 |
| 32 | 0.087 | 0.817 | -1.060 | -0.823 |
| 18 | 1.365 | 1.294 | 0.522 | -0.660 |
| 20 | 0.404 | 0.306 | 0.305 | -0.124 |
| 26 | 1.517 | 0.868 | 1.173 | -0.312 |
| 28 | 0.834 | 1.466 | 0.368 | -0.184 |
| 42 | 0.759 | 0.588 | 0.294 | -0.103 |
| 4 | 0.601 | -0.099 | -0.129 | 0.106 |
| 23 | 0.287 | 0.077 | -0.013 | 0.021 |
| 24 | 0.351 | 0.056 | 0.074 | 0.000 |
| 34 | 0.245 | 0.113 | 0.000 | 0.000 |
| 37 | -1.167 | 0.000 | 0.000 | 0.000 |

*Note.* F = Factor; ICASP = Inventory of Clinician Attitudes about Suicide Prevention.

*Table 3d. ICASP Item Response Category Scoring Coefficients*

| ICASP Item | 0 | 1 | 2 | 3 | 4 |
| --- | --- | --- | --- | --- | --- |
| 8 | 0.000 | 2.951 | 4.106 | 3.326 | 4.000 |
| 13 | 0.000 | 2.154 | 2.006 | 4.259 | 4.000 |
| 17 | 0.000 | 1.885 | 3.077 | 4.338 | 4.000 |
| 22 | 0.000 | 1.607 | 2.347 | 3.267 | 4.000 |
| 27 | 0.000 | 1.389 | 2.000 | NA | NA |
| 29 | 0.000 | 1.596 | 2.859 | 3.406 | 4.000 |
| 33 | 0.000 | 0.530 | 0.779 | 1.057 | 4.000 |
| 38 | 0.000 | 1.601 | 2.000 | NA | NA |
| 15 | 0.000 | 1.517 | 2.464 | 3.349 | 4.000 |
| 16 | 0.000 | 0.637 | 1.581 | 2.079 | 4.000 |
| 19 | 0.000 | 1.716 | 2.635 | 3.339 | 4.000 |
| 32 | 0.000 | 3.153 | 3.423 | 3.859 | 4.000 |
| 18 | 0.000 | 2.819 | 3.059 | 3.332 | 4.000 |
| 20 | 0.000 | -2.727 | -0.162 | 1.216 | 4.000 |
| 26 | 0.000 | 2.637 | 2.906 | 3.213 | 4.000 |
| 28 | 0.000 | 2.335 | 2.821 | 3.430 | 4.000 |
| 42 | 0.000 | 0.975 | 1.490 | 2.436 | 4.000 |
| 4 | 0.000 | 1.306 | 1.708 | 1.865 | 4.000 |
| 23 | 0.000 | -8.247 | -5.029 | 3.000 | NA |
| 24 | 0.000 | -1.176 | -4.540 | -2.027 | 4.000 |
| 34 | 0.000 | -6.014 | -4.703 | -2.217 | 4.000 |
| 37 | 0.000 | 1.535 | 2.000 | NA | NA |

*Note.* F = Factor; ICASP = Inventory of Clinician Attitudes about Suicide Prevention.

*Table 3e. ICASP Item Response Category Attractiveness*

| ICASP Item | 0 | 1 | 2 | 3 | 4 |
| --- | --- | --- | --- | --- | --- |
| 8 | 0.000 | 30.708 | -9.723 | 18.987 | -6.692 |
| 13 | 0.000 | 0.892 | 0.526 | -1.836 | -2.789 |
| 17 | 0.000 | 0.597 | -0.769 | -2.522 | -3.917 |
| 22 | 0.000 | 2.154 | 2.209 | 1.990 | 0.119 |
| 27 | 0.000 | 29.674 | -8.763 | NA | NA |
| 29 | 0.000 | 3.414 | 4.092 | 4.259 | 2.748 |
| 33 | 0.000 | 3.566 | 3.702 | 3.241 | -22.626 |
| 38 | 0.000 | 0.655 | -1.779 | NA | NA |
| 15 | 0.000 | 5.631 | 7.136 | 5.618 | 2.113 |
| 16 | 0.000 | 6.003 | 9.094 | 6.751 | -19.740 |
| 19 | 0.000 | 4.961 | 6.899 | 5.988 | 3.461 |
| 32 | 0.000 | 9.619 | 10.719 | 11.670 | 10.841 |
| 18 | 0.000 | 13.852 | 15.849 | 16.116 | 14.674 |
| 20 | 0.000 | -0.879 | 2.069 | 3.226 | 2.019 |
| 26 | 0.000 | 13.308 | 15.893 | 16.966 | 16.202 |
| 28 | 0.000 | 10.230 | 12.412 | 13.932 | 12.326 |
| 42 | 0.000 | 3.530 | 5.015 | 7.031 | 5.654 |
| 4 | 0.000 | 1.376 | 2.343 | 3.545 | 3.159 |
| 23 | 0.000 | 1.486 | 3.052 | 1.291 | NA |
| 24 | 0.000 | -0.103 | 1.551 | 4.390 | 4.213 |
| 34 | 0.000 | 1.743 | 3.080 | 4.216 | 1.889 |
| 37 | 0.000 | 2.516 | 1.107 | NA | NA |

*Note. Note.* F = Factor; ICASP = Inventory of Clinician Attitudes about Suicide Prevention.

**Table 4.** *Factor Correlations for ICASP Four Factor Solution.*

| Factor No. | Factor Name | 1 | 2 | 3 | 4 |
| --- | --- | --- | --- | --- | --- |
| 1 | Clinician’s Approach | --- |  |  |  |
| 2 | Clinician Avoidance | 0.156 | --- |  |  |
| 3 | Moral Rights | -0.018 | 0.020 | --- |  |
| 4 | Clinician Comfort | 0.370 | -0.041 | -0.253 | --- |

*Note*. MHP=Mental health provider. No correlations were significant between factors.

**Figure 1.** *EFA Model Item Reduction Decision Tree.*

*Note*. EFA=Exploratory factor analysis. Rectangles represent decision points; diamonds represent actions. Decision flow is repeated until model is retained.

**Figure 2**. *Parallel EFA Scree plot comparing observed, simulated, and resampled data*

*Note.* Resampled data bootstrapped 1,000 times.

**Figure 3.** *Methods for deriving number of factors*

*Note*. Results from Very Simple Structures analysis demonstrating ideal number of factors based on VSS complexity, Velicer MAP, Empirical BIC, and Root Mean Residual.

**Figure 4**. *Comparison of 5 and 6-factor solutions: Initial with all items and second run with items dropped*

*Note.* Comparing 5 and 6-factor solutions using varimax rotation. Standardized loadings shown. Only significant loadings exceeding 0.350 shown. Red dashed lines indicate significant loadings present in only the 6-factor solution.

**Appendix I. Initial ICASP Item Pool**

**Instructions:** We are interested in your perspectives about suicide because you are a mental health provider. Please select the answer choice for each statement below that most closely reflects your level of agreement. While the response options may not provide the perfect fit, we appreciate your effort to give us the best, most precise response possible.

Responses on a 0-4 scale, strongly disagree to strongly agree

0 = Strongly disagree

1 = Disagree

2 = Neither disagree or agree

3 = Agree

4 = Strongly agree

1. A client’s background and culture should be taken into consideration when assessing suicidality.
2. Treatment should prioritize managing suicidal thoughts over addressing risk and protective factors.
3. I allow a client to choose the focus of therapy when they are having suicidal thoughts.
4. It is ethical for a therapist to acknowledge a client’s wish to die by suicide.
5. I use research evidence when discussing suicide with a client.
6. There is nothing a therapist can do to prevent a client from attempting suicide.
7. My knowledge of suicide research helps me understand why a client is experiencing suicidal thoughts.
8. I am comfortable when a client raises the topic of suicide.
9. I trust that a client will communicate their suicidal thoughts to me.
10. When I talk about suicide with a client, I ask about their specific reasons for wanting to die by suicide.
11. The therapeutic alliance is essential to preventing a client from attempting suicide.
12. Treatment should focus on addressing risk and protective factors when a client is experiencing suicidal thoughts.
13. I want to refer clients to other therapists when they are at risk for suicide.
14. Suicide should be the primary focus of therapy when a client expresses suicidal thoughts.
15. It is important to respect a client’s autonomy when they are suicidal.
16. I respect a client’s decision to end their life.
17. I am more distracted in sessions with suicidal clients compared to other clients.
18. A therapist should always instill hope when a client is experiencing suicidal thoughts.
19. A client has the moral right to choose to die by suicide.
20. I have the ethical obligation as a therapist to help a client choose life over death by suicide.
21. The details of a client’s suicidal thoughts are more important to focus on compared to underlying causes.
22. If a client were to die by suicide, I would feel personally responsible.
23. The therapeutic alliance is a strong protective factor in preventing a client’s suicide.
24. A strong relationship between the client and therapist facilitates discussion of their suicidal thoughts.
25. A client should be empowered to take control of their treatment when experiencing suicidal thoughts.
26. I always encourage a client to choose life over suicide.
27. I remain in the moment when working with clients experiencing suicidal thoughts.
28. I feel a need to protect my client’s life when they have suicidal thoughts.
29. I am anxious about legal liability when a client tells me they have suicidal thoughts.
30. Underlying causes are more important to address than the specific suicidal thoughts themselves.
31. A strong relationship with a suicidal client does not guarantee they will disclose their suicidal thoughts.
32. It is ultimately a client’s decision to live or die when they are having suicidal thoughts.
33. I feel anxious working with suicidal clients.
34. The relationship between a client and therapist may prevent a client from attempting suicide.
35. A client’s personal experience is more important than research evidence in understanding their suicidal thoughts.
36. A client must take a proactive role in treatment for their suicidal thoughts.
37. Building rapport with a client will not reduce the chances a client dies by suicide.
38. I am not afraid to talk about suicide with clients.
39. Current research is more important than a client’s personal experience in understanding their suicidal thoughts.
40. It is my job as a therapist to prevent a client’s suicide.
41. It is important to focus on the source of the distress when a client is having suicidal thoughts.
42. It is my job as a therapist to communicate alternatives to suicide to a client.
43. I understand a client’s reason for wanting to die by suicide based on the details of their suicidal thoughts.
44. I am legally responsible for a suicidal client’s life.
45. A client is responsible for their own life.
46. It is important to prioritize suicidal thoughts during therapy when a client is suicidal.
47. It is helpful to be aware of the current research on suicide to help clients experiencing suicidal thinking.
48. I cannot dissuade a client from attempting suicide.

**Appendix II. Final ICASP with Scoring Instructions**

**Inventory of Clinician Attitudes about Suicide Prevention (I-CASP)**

**Instructions:** We are interested in your perspectives about suicide because you are a mental health provider. Please select the answer choice for each statement below that most closely reflects your level of agreement. While the response options may not provide the perfect fit, we appreciate your effort to give us the best, most precise response possible.

Responses on a 0-4 scale, strongly disagree to strongly agree

0 = Strongly disagree

1 = Disagree

2 = Neither disagree or agree

3 = Agree

4 = Strongly agree

1. It is ethical for a therapist to acknowledge a client’s wish to die by suicide.
2. I am comfortable when a client raises the topic of suicide.
3. I want to refer clients to other therapists when they are at risk for suicide.
4. It is important to respect a client’s autonomy when they are suicidal.
5. I respect a client’s decision to end their life.
6. I am more distracted in sessions with suicidal clients compared to other clients.
7. A therapist should always instill hope when a client is experiencing suicidal thoughts.
8. A client has the moral right to choose to die by suicide.
9. I have the ethical obligation as a therapist to help a client choose life over death by suicide.
10. If a client were to die by suicide, I would feel personally responsible.
11. The therapeutic alliance is a strong protective factor in preventing a client’s suicide.
12. A strong relationship between the client and therapist facilitates discussion of their suicidal thoughts.
13. I always encourage a client to choose life over suicide.
14. I remain in the moment when working with clients experiencing suicidal thoughts.
15. I feel a need to protect my client’s life when they have suicidal thoughts.
16. I am anxious about legal liability when a client tells me they have suicidal thoughts.
17. It is ultimately a client’s decision to live or die when they are having suicidal thoughts.
18. I feel anxious working with suicidal clients.
19. The relationship between a client and therapist may prevent a client from attempting suicide.
20. Building rapport with a client will not reduce the chances a client dies by suicide.
21. I am not afraid to talk about suicide with clients.*
22. It is my job as a therapist to communicate alternatives to suicide to a client.

**Scoring instructions**:

Clinician Approach: Sumer items 1, 7, 9, 11, 12, 13, 15, 19, 22

Clinician Avoidance: *Reverse code item 21, then sum items 3, 6, 10, 16, 18, 20, 21
Moral Rights: Sum items 4 5, 8, 17

Clinician Comfort: Sum items: 2, 14
